# Transcriptome Profiling of a Multiple Recurrent Muscle-Invasive Urothelial Carcinoma of the Bladder by Deep Sequencing

**DOI:** 10.1371/journal.pone.0091466

**Published:** 2014-03-12

**Authors:** Shufang Zhang, Yanxuan Liu, Zhenxiang Liu, Chong Zhang, Hui Cao, Yongqing Ye, Shunlan Wang, Ying'ai Zhang, Sifang Xiao, Peng Yang, Jindong Li, Zhiming Bai

**Affiliations:** 1 Affiliated Haikou Hospital, Xiangya School of Medicine Central South University, Haikou Municipal People's Hospital, Haikou, China; 2 Department of Genetic Disease, the First Affiliated Hospital of Xinxiang Medical University, Xinxiang, China; 3 Department of Shanghai Claison Bio-Technology, Shanghai, China; Eberhard-Karls University, Germany

## Abstract

Urothelial carcinoma of the bladder (UCB) is one of the commonly diagnosed cancers in the world. The UCB has the highest rate of recurrence of any malignancy. A genome-wide screening of transcriptome dysregulation between cancer and normal tissue would provide insight into the molecular basis of UCB recurrence and is a key step to discovering biomarkers for diagnosis and therapeutic targets. Compared with microarray technology, which is commonly used to identify expression level changes, the recently developed RNA-seq technique has the ability to detect other abnormal regulations in the cancer transcriptome, such as alternative splicing. In this study, we performed high-throughput transcriptome sequencing at ∼50× coverage on a recurrent muscle-invasive cisplatin-resistance UCB tissue and the adjacent non-tumor tissue. The results revealed cancer-specific differentially expressed genes between the tumor and non-tumor tissue enriched in the cell adhesion molecules, focal adhesion and ECM-receptor interaction pathway. Five dysregulated genes, including CDH1, VEGFA, PTPRF, CLDN7, and MMP2 were confirmed by Real time qPCR in the sequencing samples and the additional eleven samples. Our data revealed that more than three hundred genes showed differential splicing patterns between tumor tissue and non-tumor tissue. Among these genes, we filtered 24 cancer-associated alternative splicing genes with differential exon usage. The findings from RNA-Seq were validated by Real time qPCR for CD44, PDGFA, NUMB, and LPHN2. This study provides a comprehensive survey of the UCB transcriptome, which provides better insight into the complexity of regulatory changes during recurrence and metastasis.

## Introduction

The bladder cancer is the seventh most prevalent type of cancer worldwide. Global estimates suggest that in 2008, approximately 386,300 new bladder cancer cases were diagnosed and that 150,200 patients succumbed to the disease [1]. As the major subtype of bladder cancer, urothelial carcinoma of the bladder (UCB) is the fifth most expensive cancer to treat, accounting for $3.7 billion in direct costs in 2001 [2]. The costs are high because most patients survive long term, recurrence is frequent and lifelong surveillance is required. This disease occurs predominantly in men, yet it is increasing in incidence among women in a manner that cannot be entirely explained by increased tobacco use [3].

Approximately 80% of bladder cancers present as non-muscle invasive urothelial carcinoma, 70% of them will recur, and 10–20% of them will progress and invade the bladder muscle [4]. Of the patients initially presenting with muscle-invasive UCB, 50% will relapse with metastatic disease [5], [6]. High-grade muscle-invasive disease represents a life-threatening condition and requires timely treatment [7], [8].

Prior studies of genomic alterations have revealed that somatic changes, including point mutations [9], [10], DNA rearrangements (reviewed in [6]) and copy number variations [11] [12], can result in mutations that drive the development of UCB. As a consequence of changes in the cancer genome, the reprogramming of the transcriptome leads to abnormal cellular behavior and thus directly contributes to cancer progression [13], [14]. Studying the cancer transcriptome not only enables us to fill in the gap between driver mutations and cancer cell behavior, but also allows us to identify additional candidate cancer-related mutations and the molecular basis of gene regulation [14].

Alternative splicing (AS), the process by which splice sites are differentially utilized to produce different mRNA isoforms, is a key component in expanding a relatively limited number of genes into very complex proteomes in metazoans. Several evidences suggested that AS changes were associated with cancers [15], [16], [17]. The cancer-specific splice variants may potentially be used as diagnostic, prognostic, and predictive biomarkers as well as therapeutic targets [18].

The recent development of massively parallel sequencing (RNA-seq) provides a powerful approach to profile the transcriptome with greater efficiency and higher resolution [19]. The advantage of RNA-seq is that this technique makes feasible the study of the cancer transcriptome complexity, including alternative splicing, isoform usage, gene fusions and novel transcripts (reviewed in [20], [21]. Despite the prevalence of using RNA-seq to study various cancer transcriptomes [20], the deep annotation of UCB gene expression profiling has not been performed.

In this study, we aimed to thoroughly annotate the transcriptomes of UCB tissue and adjacent non-tumor tissue from a single recurrent and cisplatin-resistance patient by RNA-seq. First, we found several dysregulated genes. Second, we performed the enrich analysis of Gene Ontology (GO) and pathway analysis of the dysregulated genes. Third, we investigated the differential splicing pattern between tumor and non-tumor tissue, and found out the cancer-associated genes with different exon skipping events. Finally, to validate our sequencing results, quantitative real-time PCR (qPCR) was used to confirm the difference of gene expression and the differential usage of splice variants in the sequencing patient and eleven additional patients.

## Results

### Analysis of RNA-Seq data

Two samples — UCB tissue (stage II, multiple recurrent and cisplatin-resistance) and distant non-tumor tissue — were collected from a Chinese male patient. Fig. S1 showed the pathological diagnostic images of the UCB tissue. All samples were subjected to massively parallel paired-end cDNA sequencing. In total, we obtained 32.0 million and 31.4 million read pairs from the UCB and non-tumor tissue, respectively. We used TopHat to align the reads to the UCSC (the University of California Santa) reference human genome Hg19. The uniquely aligned reads for the two samples ranged from 26.4 million to 28 million pairs. The proportion of reads that mapped to the Ensembl reference genes was ∼78% for the both samples. The average coverage of our sequencing depth was approximately 50 times of human transcriptome (approximately 113 millon bp, based on the total length of the uniquely annotated exon region in the Ensembl database). In addition, only ∼1% reads were mapped to rRNA, indicating that our libraries are properly constructed and faithfully represent the expression of genes with ploy (A). The details of the mapping results are listed in Table 1.

**Table 1:** Statistics of bladder cancer transcriptome mapping to human genome Hg19.

	Tumor	Non-tumor
**Total reads**	62,822,760 (100%)	63,977,860 (100%)
**Uniquely Mapped Single Reads**	3,143,012 (5.0%)	5,226,940 (8.2%)
**Uniquely Mapped Paired Reads**	52,859,962 (84.1%)	47,634,322 (74.5%)
**Total Uniquely Mapped Reads**	56,002,974 (89.1%)	52,861,262 (82.6%)
**Uniquely Splice Junction Reads**	10,935,040 (17.4%)	12,952,784 (20.2%)
**Total Uniquely Mapped length (bp)**	5,749,364,918(51x^#^)	5,462,298,875 (48x^#^)

#: Sequencing coverage on human transcriptome (approximately 113 million bps which was estimated as the total length of all unique exons according to Ensembl database).

### Analysis of differentially expressed genes

After mapping the RNA-Seq reads to the reference genome with TopHat, transcripts were assembled and their relative abundances were calculated using Cufflinks [22]. The Cufflinks use Cuffdiff algorithm to measure the gene expression and to identify the differentially expressed genes (DEGs). The normalized expression level of each gene was measured by Fragments Per Kilobase of exon per Million fragments mapped (FPKM). By requiring that the FPKM was greater than one, we detected 14,520 and 14,199 expressed genes in the tumor and non-tumor samples respectively, which included the majority of the annotated human reference genes (See Table S1 for details). The global gene expression profiles of two samples was correlated (Pearson correlation coefficient R = 0.77) (Fig. S2A). We totally detected 1879 significant DEGs (FDR<0.01, FDR: False Discovery Rate) between the two samples (Table S1). The “volcano plot” (Fig. S2B) and MA-plot (Fig. S2C) of the gene expression profiles show that the number of up- and down-regulated genes was nearly equal relative to the q-value and expression level, suggesting that the significance of the statistics test was not bias toward up- or down-regulated genes and the dysregulated genes is not biased toward highly or lowly expressed genes.

### Function enrichment analysis of differentially expressed genes

To better understand the function of DEGs, we conducted an enrichment analysis of Gene Ontology (GO) for the dysregulated genes. We performed enrichment tests for significantly dysregulated genes that were detected in the UCB and non-tumor tissue using online tools from DAVID [23]. In total, the dysregulated genes in UCB were categorized into 22 GO terms of Biological Process (Table 2, p<0.05, corrected by Bonferroni correction). Most of terms were related to immune response, cell adhesion, response to wounding, extracellular structure organization, locomotion (chemotaxis and taxis), leukocyte activation, and so on.

**Table 2:** Gene Ontology terms of enriched differentially expressed genes in bladder cancer.

GO Term in Biological Process	Fold Enrichment^#^	Corrected p value*
GO:0006955∼immune response	2.30	4.69E-18
GO:0007155∼cell adhesion	2.15	1.84E-14
GO:0022610∼biological adhesion	2.15	2.08E-14
GO:0009611∼response to wounding	1.99	4.60E-07
GO:0006952∼defense response	1.83	1.34E-05
GO:0006954∼inflammatory response	2.13	8.59E-05
GO:0002684∼positive regulation of immune system process	2.33	1.31E-04
GO:0050865∼regulation of cell activation	2.53	4.71E-04
GO:0043062∼extracellular structure organization	2.57	6.42E-04
GO:0016337∼cell-cell adhesion	2.14	9.25E-04
GO:0050863∼regulation of T cell activation	2.81	2.65E-03
GO:0030198∼extracellular matrix organization	2.94	2.66E-03
GO:0006935∼chemotaxis	2.48	3.60E-03
GO:0042330∼taxis	2.48	3.60E-03
GO:0002694∼regulation of leukocyte activation	2.39	8.66E-03
GO:0045321∼leukocyte activation	2.11	9.44E-03
GO:0050867∼positive regulation of cell activation	2.76	9.99E-03
GO:0051249∼regulation of lymphocyte activation	2.45	1.46E-02
GO:0001775∼cell activation	1.98	1.66E-02
GO:0050778∼positive regulation of immune response	2.42	2.67E-02
GO:0046649∼lymphocyte activation	2.17	3.37E-02
GO:0002252∼immune effector process	2.45	4.33E-02

#: Fold Enrichment  =  (number of differentially expressed genes with the GO term/number of differentially expressed genes)/(number of expressed genes with the GO term/number of expressed genes)

*: p value corrected by method of Bonferroni, and only GO terms of the corrected p value less than 0.05 were shown.

A more informative analysis of functional annotation can be achieved by studying the enrichment of differentially expressed genes in a particular pathway. We used DAVID [23] to analyze which KEGG pathway was enriched with dysregulated genes in UCB. The pathways enriched with DEGs are listed in Table 3 (FDR<0.05). The cell adhesion molecules (CAMs) pathway was the most significant pathway (FDR = 2.67E-08). In addition, the focal adhesion, ECM (extracellular matrix)-receptor interaction pathway, and some disease pathway were also enriched.

**Table 3:** KEGG pathways of enriched differentially expressed genes in bladder cancer.

KEGG pathway	Fold Enrichment#	FDR*
hsa04514:Cell adhesion molecules (CAMs)	3.02	2.67E-08
hsa05416:Viral myocarditis	2.81	1.63E-03
hsa04940:Type I diabetes mellitus	3.43	2.73E-03
hsa05340:Primary immunodeficiency	3.48	9.09E-03
hsa05330:Allograft rejection	3.39	1.16E-02
hsa04640:Hematopoietic cell lineage	2.32	1.74E-02
hsa05320:Autoimmune thyroid disease	2.82	1.84E-02
hsa05332:Graft-versus-host disease	3.12	2.25E-02
hsa05412:Arrhythmogenic right ventricular cardiomyopathy (ARVC)	2.33	3.30E-02
hsa04512:ECM-receptor interaction	2.24	3.52E-02
hsa04672:Intestinal immune network for IgA production	2.71	4.21E-02
hsa04510:Focal adhesion	1.71	4.36E-02

# : Fold Enrichment  =  (number of differentially expressed genes in the pathway/number of differentially expressed genes)/(number of expressed genes in the pathway/number of expressed genes)

*: False Discovery Rate provided by DAVID, only pathways of the FDR less than 0.05 were shown.

**Figure 1:**
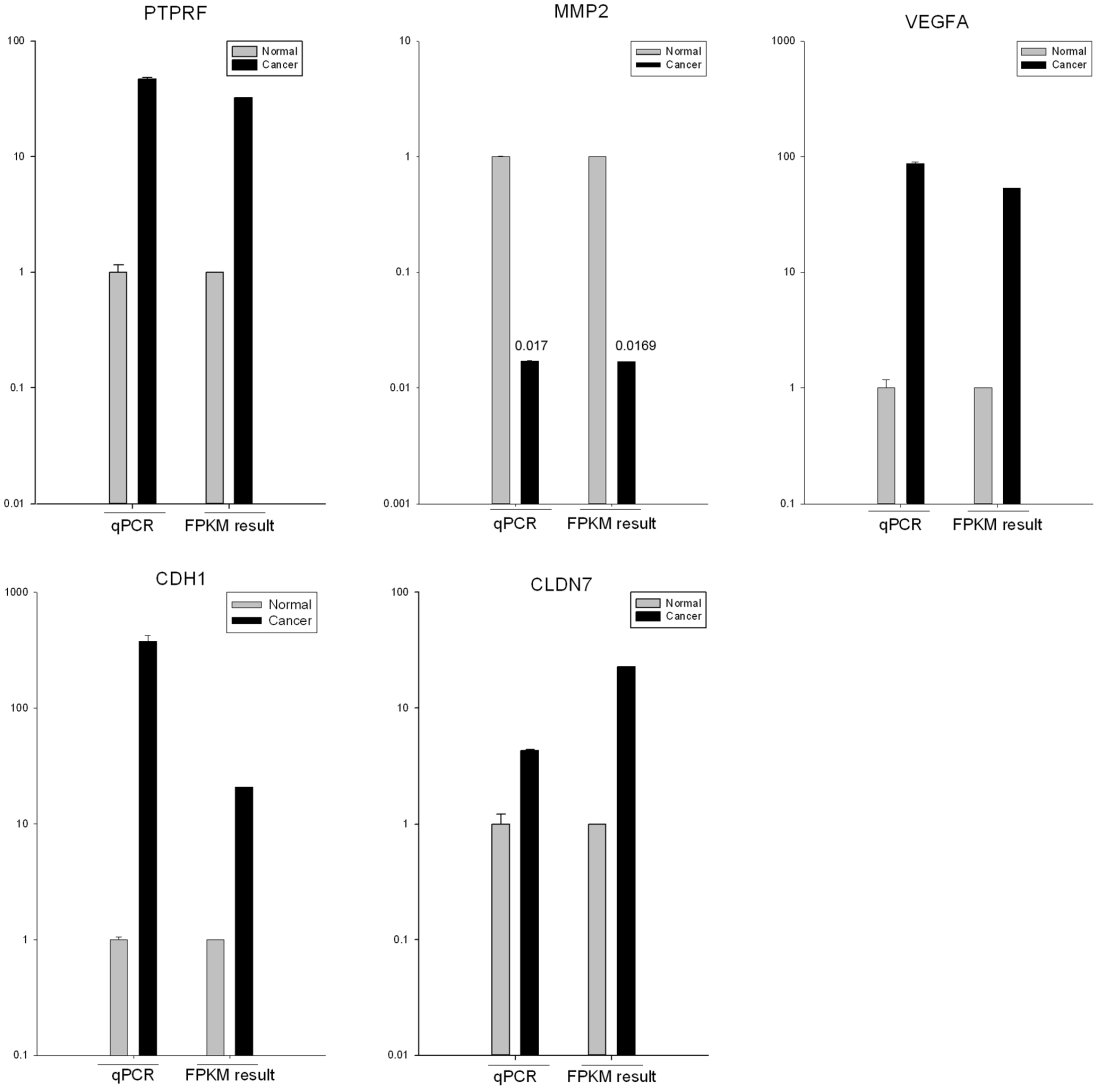
The differentially expressed genes detected by RNA-seq are confirmed by qRT-PCR. qRT-PCR was performed for five genes that are identified as differential expressed genes between UCB and non-tumor tissues. The expression level of each gene was normalized to the level in non-tumor tissue. A-E: PTPRF, MMP2, VEGFA, CDH1 and CLDN7.

To experimentally confirm the differentially expressed genes identified by RNA-seq, we performed the validation by quantitative real-time PCR (qRT-PCR). We chose five candidate genes (PTPRF, MMP2, VEGFA, CDH1 and CLDN7) that were detected differential expression by Cuffdiff (Table S2) and involved in Bladder cancer pathway, cell adhesion molecules (CAMs) pathway and focal adhesion pathway. We used GAPDH as an endogenous control in these reactions. The qRT-PCR results confirmed that all of these candidate genes expressed differently between UCB and non-tumor tissue, as shown in Fig. 1.

**Figure 2:**
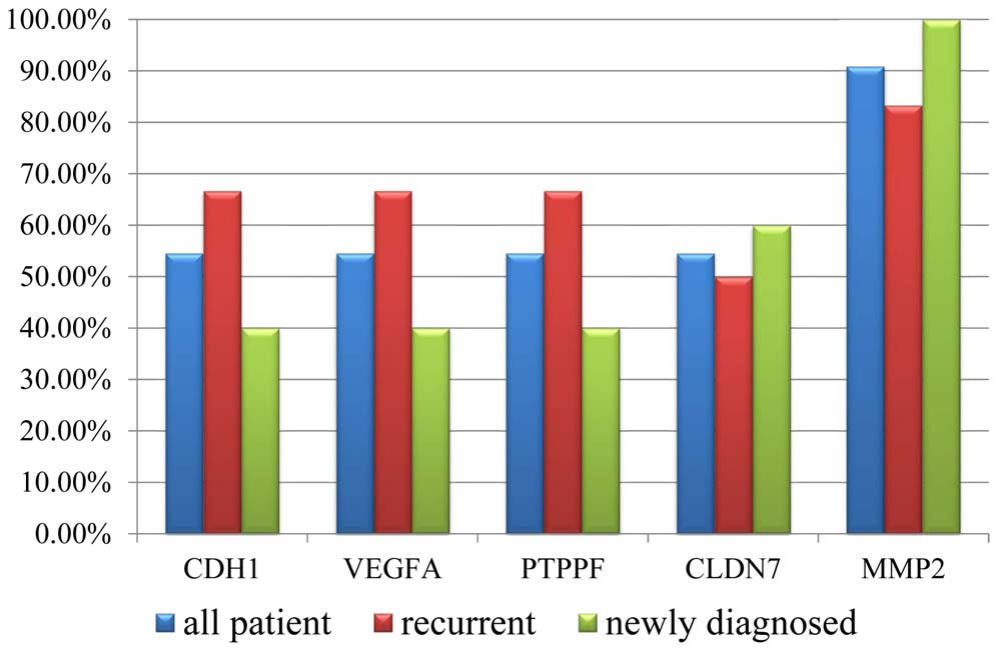
qRT-PCR validation of the differentially expressed genes in the additional patients. qRT-PCR was performed for five differentially expressed genes (CDH1, VEGFA, PTPRF, CLDN7 and MMP2) in the additional 11 patients (including 6 recurrent and drug-resistant UCB patients and 5 newly diagnosed patients). The histogram showed the proportion of validated patients in all cases (blue), the recurrent cases (red) and newly diagnosed (green).

To examine whether these genes were always dysregulated in the bladder cancer, we performed the qRT-PCR to test the expression changes for the five genes between the paired cancer and none-cancer tissue in eleven additional patients (which including 6 recurrent UCB patients and 5 newly diagnosed). The result showed that, CDH1, VEGFA, PTPRF and CLDN7 were up-regulated in six cancer samples, and MMP2 was down-regulated in ten cancer samples, suggested that these genes, especially MMP2, were dysregualted in most UCB samples (Table S3). And we also found that CDH1, VEGFA, PTPRF were up-regulated in 66.7% (4/6) recurrent patients but only 40% (2/5) newly diagnosed patients (Fig. 2), suggesting the three genes might associated with the recurrence of UCB.

### Alternative splicing events in bladder cancer

One gene locus can express multiple isoforms by alternative splicing (AS). The transcript diversity leads to plastic transcriptional networks in cancer, which are important to generate the unusual properties of cancer cells [17], [24]. We thus perform genome-wide screening to identify the cancer-restricted alternative splicing events using software MISO (the Mixture of Isoforms) [25]. In total, we detected 25,695 and 23,769 alternative splicing events in the UCB and non-tumor tissue, respectively (Table 4). These events included seven different patterns: alternative 3′splice sites (A3SS), alternative 5′splice sites (A5SS), alternative first exons (AFE), mutually exclusive exons (MXE), retained introns (RI), skipped exons (SE) and tandem 3′UTRs (TUTR). Half of these events were exon skipping (Table 4).

**Table 4:** Statistics of alternative splicing events in bladder cancer.

	Counts		Percentage	
Pattern of alternative splicing	Tumor	Non-tumor	Tumor	Non-tumor
A3SS: Alternative 3′splice sites	3293	2950	12.8%	12.4%
A5SS: Alternative 5′splice sites	4185	3770	16.3%	15.9%
AFE: Alternative first exons	470	349	1.8%	1.5%
MXE: Mutually exclusive exons	681	657	2.7%	2.8%
RI: Retained introns	2666	2206	10.4%	9.3%
SE: Skipped exons	12697	11787	49.4%	49.6%
TUTR: Tandem 3′UTRs	1703	2050	6.6%	8.6%
TOTAL	25695	23769	100.0%	100.0%

**Figure 3:**
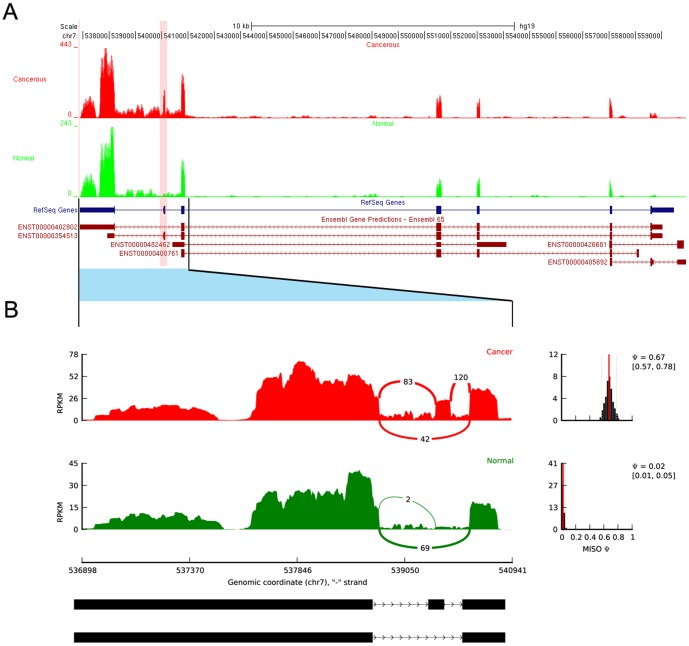
RNA-Seq read mapping to the reference gene PDGFA. A: RNA-Seq read mapping to the UCSC reference genome (hg19) of the gene PDGFA for UCB and non-tumor tissues in this study. The UCB tracks are shown in red and non-tumor tissue in green. The pink band indicated the location of skipped exon. B: The detail of junction reads mapping to the skipped exon and its neighboring exons. The Ψ (”percentage spliced in”) indicates the ratio of reads supporting inclusion exon vs. total reads supporting both inclusion and exclusion exon. The Ψ posterior distributions [25] were shown in the right side.

We next detected the differential splicing events (DSEs) between UCB and non-tumor samples using MISO (Table S4 “raw”). We found 462 DSEs from 390 unique genes, and more than half of DSEs belong to skipped exon (Table 5). We defined the genes with DSEs as differential splicing genes (DSGs). To identify reliable DSGs associated with cancer, we further filtered the DSEs by a series steps (Materials and Methods) and obtained 43 reliable DSEs from 38 unique cancer-associated DSGs (Table S4 “cancer-associated”). Of these DSEs, 25 events from 24 DSGs belong to splicing pattern “skipped exon” (Table 6). As an example, Fig. 3 showed the coverage of reads of PDGFA in the differential exon usage. The ratio of junction-reads number for the exon inclusion versus the exon exclusion was obviously higher in the cancer tissue than that in the non-tumor tissue for both of the two genes. Since the skipped exon is the most common way to generate protein products with alternative functions by truncating the functional domain in mammals [17], we focus on the analysis of differential splicing events of skipped exon in the future steps.

**Table 5:** Statistics of differential splicing genes in bladder cancer.

Pattern of alternative splicing	# of differential splicing events (percentage)	# of unique differential splicing genes (percentage)
A3SS: Alternative 3′splice sites	49(10.61%)	47(12.05%)
A5SS: Alternative 5′splice sites	52(11.26%)	46(11.79%)
AFE: Alternative first exons	14(3.03%)	10(2.56%)
MXE: Mutually exclusive exons	14(3.03%)	13(3.33%)
RI: Retained introns	101(21.86%)	95(24.36%)
SE: Skipped exons	232(50.22%)	203(52.05%)
TUTR: Tandem 3′UTRs	0(0.00%)	0(0.00%)
TOTAL	462(100.00%)	390(100.00%)

**Table 6:** Differential exon skipping events in cancer-associated genes.

Gene symbol	location of skipped exon	Ψ1&	Ψ2&	diff*	Bayes factor$	Gene description
MACF1	chr1:39946592–39946702	0.81	0.1	0.71	5.90E+275	microtubule-actin crosslinking factor 1
CTNND1	chr11:57583387–57583473	0.46	0.01	0.45	1.70E+244	catenin (cadherin-associated protein), delta 1
PDGFA	chr7:540068–540136	0.67	0.02	0.65	7.10E+219	platelet-derived growth factor alpha polypeptide
LPHN2	chr1:82452585–82452713	0.68	0.04	0.64	4.50E+216	latrophilin 2
ADD3	chr10:111892063–111892158	0.9	0.1	0.8	2.40E+184	adducin 3 (gamma)
CTNND1	chr11:57556509–57556627	0.17	0.92	−0.75	4.70E+140	catenin (cadherin-associated protein), delta 1
EIF4A2	chr3:186505197–186505373	0.9	0.48	0.42	1.50E+78	eukaryotic translation initiation factor 4A2
FAT1	chr4:187511522-187511557	0.08	0.46	−0.38	4.40E+73	FAT tumor suppressor homolog 1 (Drosophila)
CD151	chr11:834458–834591	0.19	0.49	−0.3	7.32E+37	CD151 molecule (Raph blood group)
NUMB	chr14:73745989–73746132	0.36	0.03	0.33	1.53E+34	numb homolog (Drosophila)
PACSIN2	chr22:43272894–43273016	0.67	0.97	−0.3	8.69E+28	protein kinase C and casein kinase substrate in neurons 2
FNBP4	chr11:47747289–47747388	0.77	0.24	0.53	5.61E+16	formin binding protein 4
TRIM37	chr17:57094657–57094785	0.41	0.91	−0.5	5.58E+15	tripartite motif containing 37
ACTB	chr7:5569166–5569364	0.78	0.33	0.45	1.00E+12	actin, beta
NIN	chr14:51223210–51225348	0.1	0.89	−0.79	1.00E+12	ninein (GSK3B interacting protein)
THSD1	chr13:52960163–52960321	0.33	0.91	−0.58	6.60E+09	thrombospondin, type I, domain containing 1
ELK1	chrX:47509320–47509425	0.75	0.38	0.37	3.03E+08	ELK1, member of ETS oncogene family
CD44	chr11:35231512–35231601	0.96	0.59	0.37	3.33E+07	CD44 molecule (Indian blood group)
GAS8	chr16:90102041–90102095	0.37	0.74	−0.37	2.20E+06	growth arrest-specific 8
TNC	chr9:117808689–117808961	0.43	0.85	−0.42	6.28E+05	tenascin C
GSK3B	chr3:119562102–119562200	0.95	0.42	0.53	5.30E+05	glycogen synthase kinase 3 beta
UBE2V1	chr20:48700666–48700791	0.64	0.97	−0.33	3.46E+04	ubiquitin-conjugating enzyme E2 variant 1
GTF2H1	chr11:18347494–18347700	0.04	0.38	−0.34	4.91E+03	general transcription factor IIH, polypeptide 1, 62kDa
ZMYND8	chr20:45841287–45841370	0.9	0.5	0.4	3.19E+03	zinc finger, MYND-type containing 8
CIRBP	chr19:1273493–1273714	0.49	0.88	−0.39	2.44E+03	cold inducible RNA binding protein

& : Ψ, percentage spliced in, denotes the fraction of mRNAs that represent the inclusion isoform; Ψ1: Ψ in cancer sample, Ψ2: Ψ in non-tumor sample.

*: The “diff” is provided by the MISO, and indicated the degree of splicing difference between samples. It was in [−1, 1]. The positive “diff” value means that the exon was skipped more in the non-tumor tissue than that in the cancer tissue, and the negative values means the exon skipped more in the cancer tissue.

$ : The “bayes factor” provided by MISO indicate the significance of the splicing difference. It was in [0, +∞), and it was greater, then the difference was more significant.

**Figure 4:**
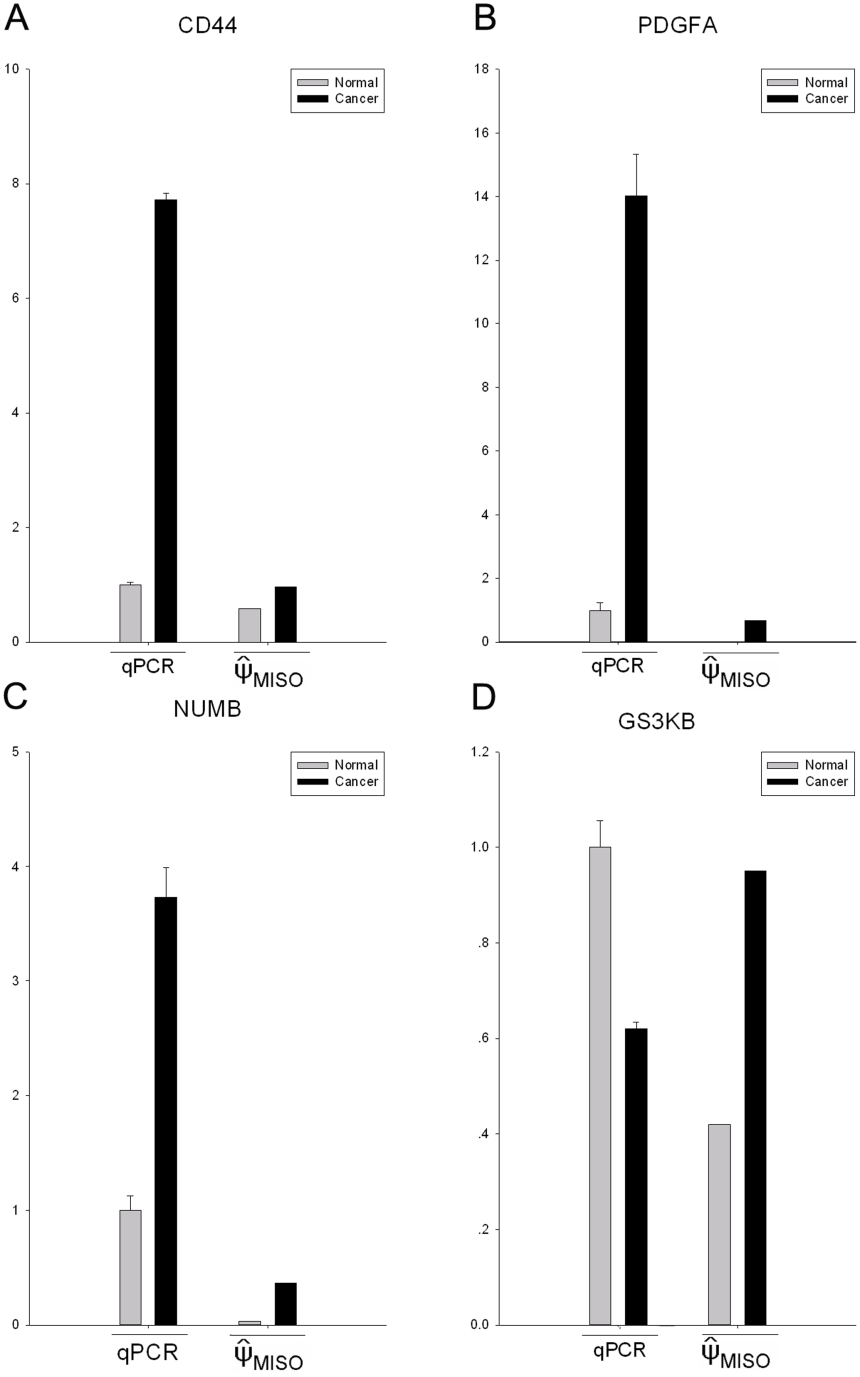
The qRT-PCR validation of differential splicing events detected by RNA-seq. qRT-PCR was performed for four genes that are identified as differential splicing genes between UCB and non-tumor tissues. The result of qRT-PCR is the relative expression level of the skipped exon and the neighboring constitutive exon. The expression level of each exon was normalized to the level in non-tumor tissue. The ΨMISO was the result of MISO, indicates the ratio of reads supporting inclusion exon vs. total reads supporting both inclusion and exclusion exon. A∼D: CD44, PDGFA, NUMB and GSK3B.

To experimentally confirm the skipped-exon DSGs identified by RNA-seq, the relative expression levels between skipped exons and their neighboring exon of selected genes were measured in the UCB and non-tumor sample by quantitative real-time PCR (qRT-PCR). We chose four candidate genes involved in KEGG pathways, including the CD44, GSK3B, PDGFA and NUMB, from above 24 differential splicing genes from MISO (the primers shown in Table S2). We used GAPDH as an endogenous control in these reactions. The result showed that except for GSK3B, another three genes, including CD44, PDGFA and NUMB, were validated (Fig. 4).

**Figure 5:**
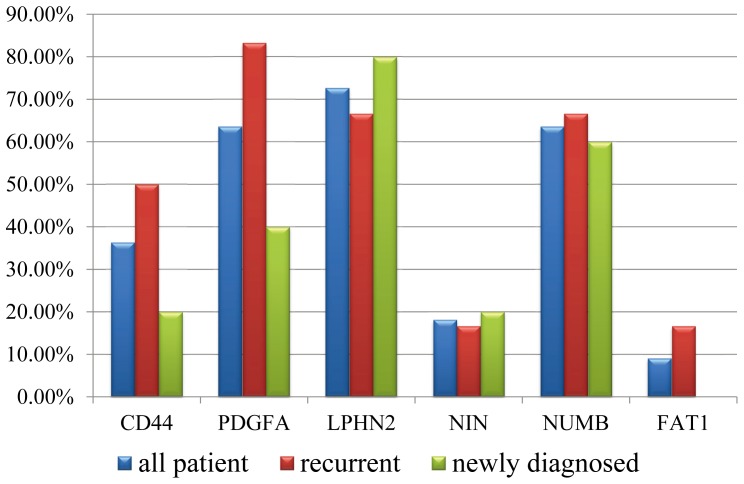
qRT-PCR validation of differential splicing events in the additional patients. qRT-PCR was performed for six differential splicing genes (CD44, PDGFA, NUMB, LPHN2, NIN and FAT1) in the additional 11 patients (including 6 recurrent and drug-resistant UCB patients and 5 newly diagnosed patients). The histogram showed the proportion of validated patients in all cases (blue), the recurrent cases (red) and newly diagnosed (green).

We next chose six differential splicing genes (CD44, PDGFA, NUMB, LPHN2, NIN, FAT1) to perform qRT-PCR validation in the eleven additional patients used in differentially expressed gene validation (Table S5). The result showed that CD44 (36%, 4/11), PDGFA (64%, 7/11), NUMB (64%, 7/11) and LPHN2 (73%, 8/11) showed exon increased exon inclusion in considerable number of UCB patients, but few patients showed the increased exon exclusion in gene NIN (18%, 2/11) and 9% (1/11). We also found that PDFGA showed increased exon inclusion in 83% (5/6) recurrent UCB samples, but only 40% (2/5) newly diagnosed samples (Fig. 5). And CD44 also showed higher proportion of exon inclusion in the recurrent samples (50%, 3/6) than that newly diagnosed (20%, 1/5). It suggested that the increased exon inclusion PDGFA (chr7∶540068-540136) and CD44 (chr11∶35231512-35231601) might associated with the recurrence of UCB.

### Bioinformatics prediction of gene fusion events

We used two algorithms, deFuse [26] and TopHat-Fusion [27], to detect gene fusion based on the pair-ends reads in the two samples. Although various results were generated by deFuse and TopHat-Fusion (Table S6), however, none reliable fusion transcript was found by manually checking the reads mapping to the fusion sequence (Methods).

## Discussion

Our study provides the first comprehensive insight into the transcriptome of a recurrent, drug-resistant and muscle-invasive urothelial carcinoma of the bladder with RNA-Seq. In total, approximately 60 million reads were generated per sample, which enabled us to quantify the gene expression abundance at a wide range [28]. The percentage of uniquely reads mapping, approximated uniform coverage in each gene (Fig. S3) and the number of expressed genes (FPKM>0) revealed that the data satisfied the quality standards of the RNA-seq and represented the majority of the transcriptome. We identified the levels of differentially expressed genes and alternative splicing patterns associated with cancer.

### Differentially expressed genes in UCB

In this study, we sampled cancer and distant non-tumor tissue from a single individual to conduct transcriptome comparisons. To determine whether our findings were in agreement with previously reported results, we systematically compared the changes in the expression of specific UCB-related genes.

We found that the vascular endothelial growth factor A (VEGFA), a member of the PDGF/VEGF growth factor family that promotes angiogenesis through nitric oxide synthase, was significantly up-regulated in UCB in cancer tissue compared to non-tumor tissue. Our result is coincident with the recent two studies using microarrays and digital gene expression profile, which both found the up-regulation of VEGFA in a large number of UCB patients [29], [30], suggesting that VEGFA might be a commonly over-expressed gene in UCB.

We found that most of matrix metalloproteinases (MMPs), especially MMP2 and MMP9, is down-regulated in cancer tissues compared to non-tumor tissue. The MMPs activate basic and acidic fibroblast growth factors (bFGF and aFGF, respectively), which in turn restimulate the MMPs to promote endothelial cell migration [31]. MMPs also stimulate scatter factor (SF), which stimulates angiogenesis. High levels of MMP-2 and MMP-9 have been associated with increasing stage and grade of UCB [32], [33], and MMP2 overexpression can predict poor relapse-free and disease-specific survival [34]. However, in the recent two studies, MMP2 was reported under-expression in UCB [29], [30].

We also detected some biological markers in the diagnosis of recurrent bladder cancer was dysregulated in UCB, including KRT20 (Cytokeratin 20) [35], BIRC5 (Survivin) [36], [37], CDH1 (E-cadherin) [38] and PSCA (Prostate Stem Cell Antigen-14) [39], [40]. The investigation in DEGs showed that our findings from RNA-Seq agreed with previous reports.

In addition, several known driver factors that are frequently mutated in UCB, including ARPC5 (p16) [41] and FGF2 [42], showed no change in expression in this study, suggesting that the genetic heterogeneity of UCB or the mutated products might be deleterious even if the expression level is unaffected.

The bladder cancer is characterized by chemoresistance although the mechanism is still not entirely known [43]. The UCB sample used in this study was diagnosed as the cisplatin resistance. We investigated the expression of the drug-resistant genes mentioned by Köberle et al., which listed the genes with cisplatin-based resistance in bladder cancer [44]. We found that genes associated with DNA repair and apoptosis pathway were dysregulated in UCB samples (Table S7), suggesting that the chemoresistance of this cancer sample might be associated with the increased DNA repair and suppression of apoptosis.

In this study, the CAMs pathway is the most significant pathway enriched with DEGs, this result confirmed with the previous report that the CAMs is common pathway enriched with DEGs in carcinomas of the bladder, kidney and testis [30]. Aberration of the CAMs pathway and ECM receptors enables cancer cells to escape their primary tumor masses, invade adjacent tissues and colonize elsewhere [45], [46]. Additionally, as demonstrated in our study, frequent deregulation of the cytokine-related pathways as well as the immune and inflammatory response processes is another common hallmark of human cancer [47]. For many solid tumors, cytokines, together with CAMs, play important roles in the induction of antitumor immune responses and tumor rejection in the tumor microenvironment where immune and malignant cells interact [48]. Moreover, recent emerging data suggested that cancer-related inflammation contributes to the proliferation and survival of tumor cells and linked this inflammation to the therapeutic response and prognosis of cancer patients [49].

### Cancer-associated differential exon skipping events

Alternative regulation of gene expression can be achieved by transcriptional and post-transcriptional regulation. The first class of dysregulation of UCB at the transcriptional level has been well studied using microarray technology [50], [51], [52]. Quantifying the second class of regulatory change remains challenging despite the invention of the exon array [53]. RNA-seq technology enables the simultaneous study of these two different mechanisms [19], [22], [54], [55]. In this study, we also investigated the second class of transcriptional dysregulation by analyzing the alternative splicing in UCB.

We performed the analysis by MISO, a probabilistic framework to quantitate the expression level of alternatively spliced genes from RNA-Seq data, and identifies differentially regulated isoforms or exons across samples [25]. By adopting the more stringent cut-off and crossing with known cancer-associated genes, we find 24 highly reliable cancer-associated differential splicing genes.

Some splicing events have been reported to be related to bladder cancer using exon arrays, including CD44, CLSTN1 and CTNND1 [53]. CD44 is a transmembrane glycoprotein that participates in many cellular processes including regulation of cell division, survival, migration, and adhesion [56]. Its splice variant CD44E (exon v8-10 expressed) can serve as a prognostic predictor and indicator of disease extent in patients with urothelial cancer [57], [58], [59]. In our study, the variant exon v8, v9 and v10 expressed in the UCB tissue, but not in the non-tumor tissue (Fig. S4), suggesting that CD44E was cancer-specific. Our result supported the CD44E as a marker in the bladder cancer diagnose.

Some cancer-associated DSGs in our result were reported in the other cancers but not reported in the bladder cancer yet, such as PDGFA, MACF1, ADD3 and NUMB. PDGFA, a member of platelet-derived growth factor family, have two isoforms corresponding to a long (PDGFAA) and a short (PDGFAB) form due to alternative splicing of exon 6 [60]. In this study, the exon 6 was skipped in non-tumor tissue but not in the cancer tissue (Fig. 3), which means that the long isoform PDGFAA was mainly expressed in cancer tissues and the short isoform PDGFAB expressed in non-tumor tissue. PDGFAA has a basic carboxy-terminal tail encoded by exon 6, attaching it to the extracellular matrix whereas PDGFAB is freely diffusible in the extracellular fluid since it lacks this retention motif [61], [62]. The role of the basic extension in PDGFAA and how it makes the long form functionally different from the short remain unknown. Expression of the long form of PDGFA was originally identified in tumor cells [60], [63], [64], and PDGFAA was cloned from a human glioma cell line [63]. The different expression of long isoforms of PDGFA was also reported in gliosarcomas of mouse [65] and liver cancer of rat [66]. A recent study showed that the long isoform of PDGFA overexpressed in the brain abnormalities and glioma-Like lesions in astrocytic cells in mice, and induced accumulation of immature cells in the mouse brain [65]. Further investigations are needed to understand the particular mechanism of the long isoform of PDGFA in UCB.

The AS events in MAFC1, ADD3 and NUMB were reported in the two recent researches in non-small cell lung cancer [67], [68]. MAFC1 belongs to the plakin family of cytoskeletal linker proteins which form bridges between different cytoskeletal elements by specialized modular domains. Using exon array and qPCR validation, Misquitta-Ali et al. found that the exon number 8 (Ensembl exon id: ENSE00001770152) of MACF1 was expressed in non-small lung cancer and breast cancer but not in the pair-matched normal tissues [68]. Our study found this AS change between the UCB and the paired non-tumor tissue (Fig. S5), suggesting that the increased exon inclusion of exon 8 of MAFC1 might be common in these cancers. MACF1 has no direct relation with cancer, it has been reported to function in the Wnt signaling pathway and to be associated with a complex containing Axin, beta-catenin, glycogen synthase kinase 3 (GSK3B), and adenomatous polyposis coli (APC) [69], which have been linked to tumorigenesis [70], [71]. Based on this, the increased inclusion of the alternative exon in MACF1 transcripts was proposed to contribute to altered Wnt signaling in the lung and colon cancers [68], and our result expands this supposition to the bladder cancer.

ADD3 (Gamma-adducin) is s a structural constituent of the spectrin-actin cytoskeleton that contains at least 16 exons, of which exon number 15 (ENSE00000986819) is a known cassette exon of 96 bp. Langer et al. reported that the long isoform of ADD3 with inclusion of exon number 15 was specifically expressed in the non-small cell lung cancer, but the cancer-special function of the isoform is unclear [67]. Our result showed the same AS event difference between in the UCB and non-tumor tissue (Fig. S6), suggesting that the long isoform of ADD3 might be also a cancer-specific transcript in bladder cancer.

NUMB plays a role in the determination of cell fates during development. The degradation of NUMB is induced in a proteasome-dependent manner by MDM2, is a membrane-bound protein that has been shown to associate with EPS15, LNX1, and NOTCH1. The increased inclusion exon 9 (ENSE00001689532) of NUMB transcripts is a highly widespread tumor-associated AS event, which was detected by exon arrays and validated by PCR in both of two independent laboratories [67], [68]. The event was detected in 37 additional patients with lung, breast and colon cancer by Misquitta-Ali et al.[68], and in 5 of 6 patients in the study of Langer et al. [67]. In our study using RNA-Seq, the inclusion exon 9 of NUMB was also significantly increased in the UCB tissue (Fig. S7). Functional analysis in lung cancer showed that tumor-associated increases in NUMB exon 9 inclusion correlated with reduced levels of NUMB protein expression and activation of the Notch signaling pathway, an event that has been linked to tumorigenesis [68]. These findings suggested that the increased inclusion exon 9 of NUMB is supposed to be a candidate of marker in the diagnosis of multiple cancers including lung, breast, colon and bladder cancer.

There are also some differential splicing events which have not been reported to be associated with tumors, such as UCB increased exon inclusion of LPHN2 (chr1∶82452585-82452713), EIF4A2 (chr3∶186505197-186505373), FAT1 (chr4∶187511522-187511557), exon exclusion of CD151 (chr11∶834458-834591), and so on. The increased exon inclusion of LPHN2 was validated in 73% (8/11) UCB patients by qRT-PCR validation. The splicing events might be a novel alternative splicing changes associated with bladder cancer. Investigation of these events will help to understand the mechanism of tumor formation and progress. In addition, we tried to ask whether the drug-resistance was related to the differential alternative splicing due to the drug-resistance of this UCB tissue. We compared the drug-resistant genes listed in Table S7 with all DSEs we detected (listed in Table S4 “raw”). However, none of the drug-resistant genes showed the differential splicing events, suggesting that the drug-resistance might be majorly associated with the dysregulation in expression level but not in alternative splicing.

## Materials and Methods

### Patient Samples

Written informed consent from the patients were obtained, and this series of studies was reviewed and approved by Institutional Ethics Committees of Haikou Municipal People's Hospital (Haikou, China). Distant normaltissue of the urinary bladder and urothelial carcinoma of the bladder (UCB), were obtained from one 69-year-old Chinese male patient who initially suffered UCB in May 2010, and recurrently in October, November 2010 and June 2011, respectively. Partial cystectomies were performed immediately following each detection. Samples used in this study were collected in the last surgery. H&E (hematoxylin and eosin stained) slides of frozen UBC tissue with patient-matched frozen normal tissue were examined by the pathologists of this study to ensure that the tumor tissues selected had high-density cancer foci and that the normal tissues were without tumor contamination. The tumor was consisted of pure transitional epithelium carcinoma, without any atypical glandular epithelial cells or squamous epithelial cells. Besides the histology, it was observed that the tumor invaded muscle (T2), no regional lymph nodes could not be assessed (N0) and no distant metastasis (M0). According TNM classification of carcinomas of the urinary bladder, the case should defined as Stage II by the International agency of research on Cancer. Tumor chemosensitivity assay reported that the tumor was resistant to common used cisplatin-based chemotherapy drugs. Fig. S1 provides the histological image of the cancerous tissue. The percentage of tumor cells in the UCB tissue was 78% by counting the relative at a 400× magnification. The additional twenty-two paired cancer and non-cancer samples using in validation were collected from six recurrent and cisplatin-resistance UCB patients (stage II) and five newly diagnosed UCB patients (stage II). All samples were independently reviewed by an additional gynecologic pathologist. The treatment histories, including chemotherapy, of cases that represent recurrence were shown in Table S8.

### Library preparation

Total RNA was extracted from non-tumor and cancerous bladder tissues with TRIzol according to the manufacturer's protocol (Invitrogen). For mRNA-seq sample preparation, the Illumina standard kit was used according to the TruSeq RNA SamplePrep Guide (Illumina). Briefly, 10 μg of total RNA from each sample was used for polyA mRNA selection using poly T oligo-conjugated magnetic beads by two rounds of purification, followed by thermal mRNA fragmentation. The fragmented mRNA was subjected to cDNA synthesis using reverse transcriptase (SuperScript II) and random primers. The cDNA was further converted into double-stranded cDNA, and after end repair (Klenow fragment, T4 polynucleotide kinase, T4 polymerase and 3-‘A’ add process [Klenow exo-fragment]), the product was ligated to Illumina Truseq adaptors. Size selection was performed using a 2% agarose gel, generating 380-bp cDNA libraries. Finally, the libraries were enriched using 15 cycles of PCR and purified with the QIAquick PCR purification kit (Qiagen). The enriched libraries were diluted with elution buffer to a final concentration of 10 nM.

### Sequencing and quality filtering

Libraries from non-tumor tissue and cancerous bladder tissue were analyzed at a concentration of 11 pM on a single Genome Analyzer *IIx* (GA*IIx*) lane using 115-bp sequencing. Raw RNA-seq data were filtered by Fastx-tools (http://hannonlab.cshl.edu/fastx_toolkit/) according to the following criteria: 1) reads containing sequencing adaptors were removed; 2) nucleotides with a quality score lower than 20 were trimmed from the end of the sequence; 3) reads shorter than 50 were discarded; and 4) artificial reads were removed. After the filtering pipeline, a total of 21.5G bp of cleaned, paired-end reads were produced.

### RNA-seq reads mapping

The clean reads were then aligned with the UCSC *H. sapiens* reference genome (build hg19) using TopHat v1.3.1[54], which initially removes a portion of the reads based on quality information accompanying each read and then maps the reads to the reference genome. The pre-built *H. sapiens* UCSC hg19 index was downloaded from the TopHat homepage and used as the reference genome. TopHat allows multiple alignments per read (up to 20 by default) and a maximum of two mismatches when mapping the reads to the reference. TopHat builds a database of potential splice junctions and confirms these by comparing the previously unmapped reads against the database of putative junctions. The default parameters for the TopHat method were used.

### Transcript abundance estimation

The aligned read files were processed by Cufflinks v1.0.3 [22], which uses the normalized RNA-seq fragment counts to measure the relative abundances of the transcripts. The unit of measurement is Fragments Per Kilobase of exon per Million fragments mapped (FPKM). Confidence intervals for FPKM estimates were calculated using a Bayesian inference method [72]. The reference GTF annotation file used in Cufflinks was downloaded from the Ensembl database (Homo_sapiens.GRCh37.63.gtf [73]). The transcript abundance data has been submitted to the GEO database with accession ID GSE33782.

### Detection of differentially expressed gene

The downloaded Ensembl GTF file was passed to Cuffdiff along with the original alignment (.SAM) files produced by TopHat. Cuffdiff re-estimates the abundance of the transcripts listed in the GTF file using alignments from the.SAM file and concurrently tests for differential expression. Only the comparisons with “q_value” less than 0.01 and test status marked as “OK” in the Cuffidff output were regarded as showing differential expression.

### Functional enrichment analysis of differentially expressed genes

The Database for Annotation, Visualization and Integrated Discovery (DAVID) v6.7 is a set of web-based functional annotation tools [23]. The unique lists of differentially expressed genes and all the expressed genes (FPKM>0 in any sample) were submitted to the web interface as the gene list and background, respectively. The cut-off of the False Discovery Rate (FDR) was set at 5%, and only the results from the GO FAT and KEGG pathways were selected as functional annotation categories for this analysis.

### Detection of differential splicing events

The Mixture of Isoforms (MISO) analysis [25] was used to detect differentially regulated exons across samples. The MISO analysis was performed according to the tool's given workflow using paired-end reads (http://genes.mit.edu/burgelab/miso/docs/). The reads alignment files (.SAM) produced by TopHat and the pre-build human genome (Hg19) alternative events downloaded from the MISO reference manual page (http://genes.mit.edu/burgelab/miso/docs/#gff-event-annotation) were used as the input. To identify highly reliable cancer-associated DES events, we filtered the DES events by the flowing steps: 1) use the stringent cuff-offs to filter the result of MISO (the absolute value of diff >0.3 and bayes factor >1000, the default cut-off of MISO were 0.2 and 10); 2) keep the genes that are overlapped with the cancer-associated gene set, which were collected from the NCBI gene database (searched by “oncogene” and “tumor suppressor gene”) and the Bushman Lab web (http://microb230.med.upenn.edu/protocols/cancergenes.html).

### Visualization of mapped reads

The mapping results were visualized using the Integrative Genomics Viewer (IGV) available at http://www.broadinstitute.org/igv/. Views of other individual genes were generated by uploading coverage.wig files to the UCSC Genome browser.

### Identifying and checking the gene fusions

All the filtered RNA-seq reads were mapped to the reference transcript sequences that were downloaded from the Ensembl database (Homo_sapiens.GRCh37.63.cdna.all.fa) using TopHat. The read pairs mapping to the same transcripts were removed, and the ends of remaining reads were truncated to maintain the 75-bp length using in-house Perl scripts. These fixed-length reads were passed to two software packages, deFuse (deFuse-0.4.2) [26] and TopHat-Fusion (TopHatFusion-0.1.0) [27], to find the candidate gene fusions. The bowtie-index used in the TopHat-Fusion was downloaded from the TopHat homepage (H. sapiens UCSC hg19). The parameters of the TopHat-Fusion used were obtained from the “Getting Started” (http://tophat-fusion.sourceforge.net/tutorial.html) tutorial. The deFuse parameters were the default settings, as described in the deFuse manual. The check of fusion transcripts was performed by mapping the reads to the identified fusion sequences. The count of unique reads spanned the fusion sites of the sequence should be greater than 5 and the reads was expected to be relatively uniformly distributed in the fusion sequences.

### Differentially expressed gene validation

The differentially expressed genes were validated by Real-Time Quantitative Polymerase Chain Reaction (RT-qPCR) using a LightCycler 480 Instrument II (Roche). The PCR volume included 10 μl sample, 5 μl 2× SYBR Green Master Mix (TOYOBO), 1 μl cDNA template and 1 pmol/μl of each oligonucleotide. The RT-qPCR thermal profile was obtained using the following procedure: 95°C for 1 min, 40 cycles of 95°C for 10 sec, 60°C for 30 sec and 72°C for 10 sec, followed by 72°C for 5 min. The program was set to reveal the melting curve of each amplicon from 60°C to 95°C and obtain a read every 0.5°C. The primer sequences are listed in Table S2. All the RT-qPCR reactions were performed in triplicate to capture intra-assay variability.

The expression levels of each target gene in the tested experimental condition (cancerous bladder tissue) were compared to the control condition ( non-tumor bladder tissue) according to Cook et al. [74]. The data were normalized using GAPDH, which had previously been identified as the best reference gene under different experimental conditions [75]. In the present analysis, GAPDH was confirmed to be stable and always showed variability less than ±1 cycle.

### Differential splicing events validation

The primers (Table S2) were designed using Primer 5 software (PREMIER Biosoft International, Palo Alto, Calif.), and The PCR experiments were performed using a Veriti Thermal Cycler (ABI). The PCR volume used comprised 10 μl sample, 1 μl 10×PCR buffer, 1 μl cDNA template, 0.2 μl dNTP, 0.2 μl Taq Enzyme (Genscript), and 0.2 pmol/μl each oligonucleotide. PCR was performed using the following procedure: 95°C for 1 min, 40 cycles of 95°C for 15 sec, 55°C for 30 sec and 72°C for 15 sec, followed by 72°C for 5 min. We confirmed the presence of the fusion gene in cancerous colon tissue. GAPDH was used as the loading control. The PCR products of the fusion gene were cloned in the pGEM-T Easy Vector (Promega) and then sequenced with the T7 primer using a 3730 DNA Analyzer (ABI).

### Data assessment

The raw sequencing data has been deposited to the NCBI Short Read Archive on accession number SRP009386.

## Supporting Information

Figure S1 **Histological image of a hematoxylin/eosin-stained section of the bladder cancer sample (original magnification ×400) (A) and distant non-tumor epithelial tissue of the urinary bladder and UCB tissues (B).**
(TIF)

Figure S2 **Differential expression analysis in the cancer and normal tissue.** A: The scatter plot for global expression between samples; the Pearson correlation coefficient is shown; B: Volcano plots for all the genes to reveal the relation between expression fold-change and q value in DEG detecting. The red and blue dots indicate that up- and down-regulated DEGs were significant at q values less than 0.01. C: MA plots for all expressed genes to reveal the relation between expression level and fold-change. Each dots stands for one gene in comparison, the dotted line in grey indicates M = 0. Differentially expressed genes were plotted in red (up-regulated) and blue (down-regulated).
(TIF)

Figure S3 **Homogeneity of reads coverage.** The genes of which FPKM>1 and cDNA length≥300 bp were assigned as three groups according to gene expression (high: the top 25%, blue; middle: the middle 50%, red; and low: the bottom 25%, green). All cDNA were divided into 100 bins, the median of reads number in each bins was shown for each group. A: Reads coverage in normal tissue; B: Reads coverage in cancer tissue.
(TIF)

Figure S4 **RNA-Seq read mapping to the reference gene CD44.** A: RNA-Seq read mapping to the UCSC reference genome (hg19) of the gene PDGFA for UCB and normal tissues in this study. The UCB tracks are shown in red and normal tissue in green. The pink band indicated the location of skipped exon. B: The detail of junction reads mapping to the skipped exon and its neighboring exons. The Ψ (”percentage spliced in”) indicates the ratio of reads supporting inclusion exon vs. total reads supporting both inclusion and exclusion exon. The Ψ posterior distributions were shown in the right side.
(TIF)

Figure S5 **RNA-Seq read mapping to the reference gene MACF1.** A: RNA-Seq read mapping to the UCSC reference genome (hg19) of the gene MACF1 for UCB and normal tissues in this study. The UCB tracks are shown in red and normal tissue in green. The pink band indicated the location of skipped exon. B: The detail of junction reads mapping to the skipped exon and its neighboring exons.
(TIF)

Figure S6 **RNA-Seq read mapping to the reference gene ADD3.** A: RNA-Seq read mapping to the UCSC reference genome (hg19) of the gene ADD3 for UCB and normal tissues in this study. The UCB tracks are shown in red and normal tissue in green. The pink band indicated the location of skipped exon. B: The detail of junction reads mapping to the skipped exon and its neighboring exons.
(TIF)

Figure S7 **RNA-Seq read mapping to the reference gene NUMB.** A: RNA-Seq read mapping to the UCSC reference genome (hg19) of the gene NUMB for UCB and normal tissues in this study. The UCB tracks are shown in red and normal tissue in green. The pink band indicated the location of skipped exon. B: The detail of junction reads mapping to the skipped exon and its neighboring exons.
(TIF)

Table S1 **Gene expression and differentially expressed genes.**
(XLS)

Table S2 **Primer sequences.**
(XLS)

Table S3 **qRT-PCR validation of five differentially expressed genes (fold change, cancer sample vs. non-cancer sample).**
(XLSX)

Table S4 **Differential splicing events.**
(XLS)

Table S5 **qRT-PCR valication of six differential splicing genes.**
(XLSX)

Table S6 **Gene fusions output by deFuse and TopHat-Fusion.**
(XLS)

Table S7 **Drug-resistant genes.**
(XLS)

Table S8 **The treatment history of cases that represent recurrence.**
(XLS)
