# Supplementary figures and images for: Transcriptome Profiling of a Multiple Recurrent Muscle-Invasive Urothelial Carcinoma of the Bladder by Deep Sequencing
Source: PLoS One. 2014 Mar 12;9(3):e91466. doi: 10.1371/journal.pone.0091466 (PMC3951401; doi:10.1371/journal.pone.0091466)

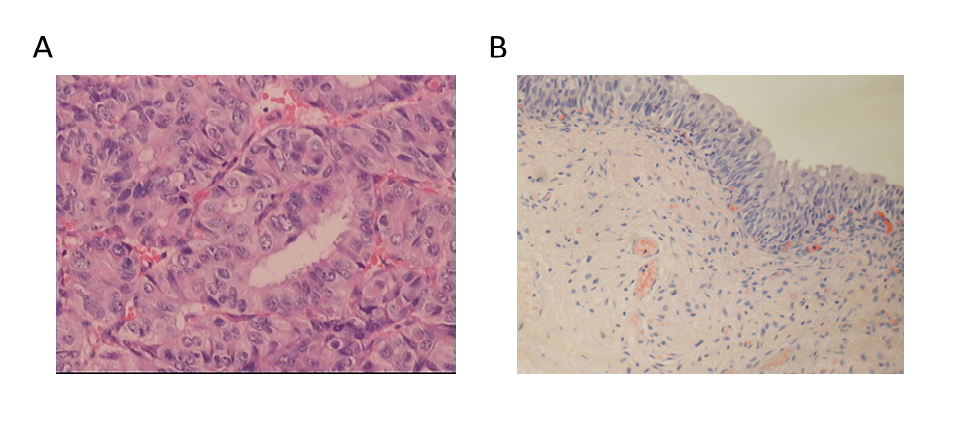

Supplement: Figure S1 — Histological image of a hematoxylin/eosin-stained section of the bladder cancer sample (original magnification ×400) (A) and distant non-tumor epithelial tissue of the urinary bladder and UCB tissues (B). (TIF) [file pone.0091466.s001.tif]

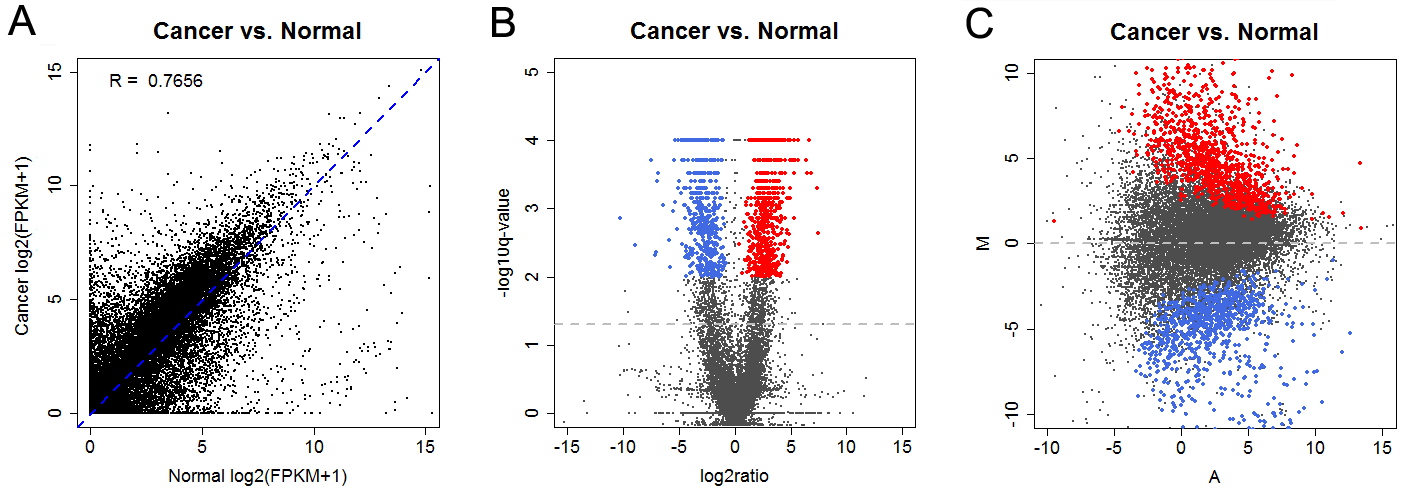

Supplement: Figure S2 — Differential expression analysis in the cancer and normal tissue. A: The scatter plot for global expression between samples; the Pearson correlation coefficient is shown; B: Volcano plots for all the genes to reveal the relation between expression fold-change and q value in DEG detecting. The red and blue dots indicate that up- and down-regulated DEGs were significant at q values less than 0.01. C: MA plots for all expressed genes to reveal the relation between expression level and fold-change. Each dots stands for one gene in comparison, the dotted line in grey indicates M = 0. Differentially expressed genes were plotted in red (up-regulated) and blue (down-regulated). (TIF) [file pone.0091466.s002.tif]

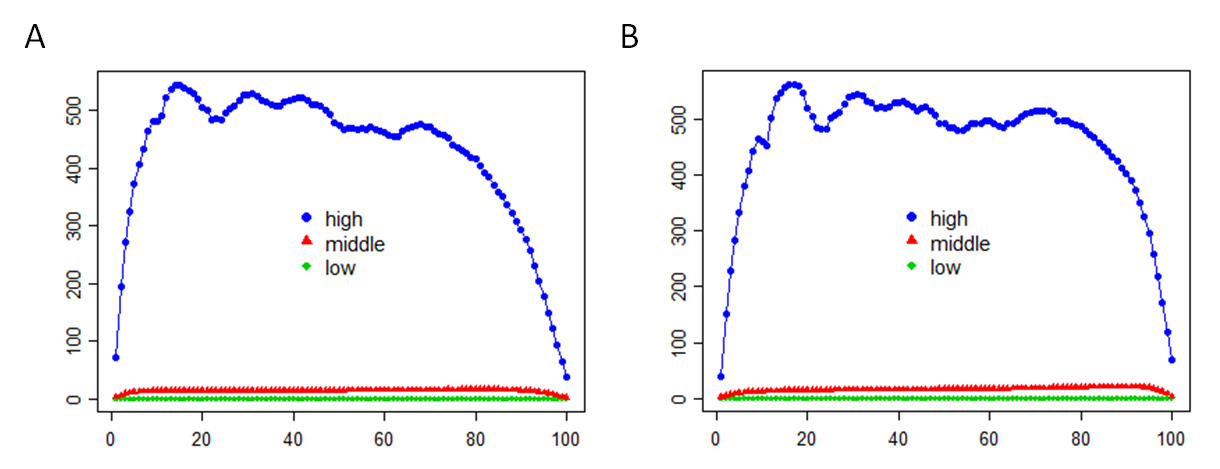

Supplement: Figure S3 — Homogeneity of reads coverage. The genes of which FPKM>1 and cDNA length≥300 bp were assigned as three groups according to gene expression (high: the top 25%, blue; middle: the middle 50%, red; and low: the bottom 25%, green). All cDNA were divided into 100 bins, the median of reads number in each bins was shown for each group. A: Reads coverage in normal tissue; B: Reads coverage in cancer tissue. (TIF) [file pone.0091466.s003.tif]

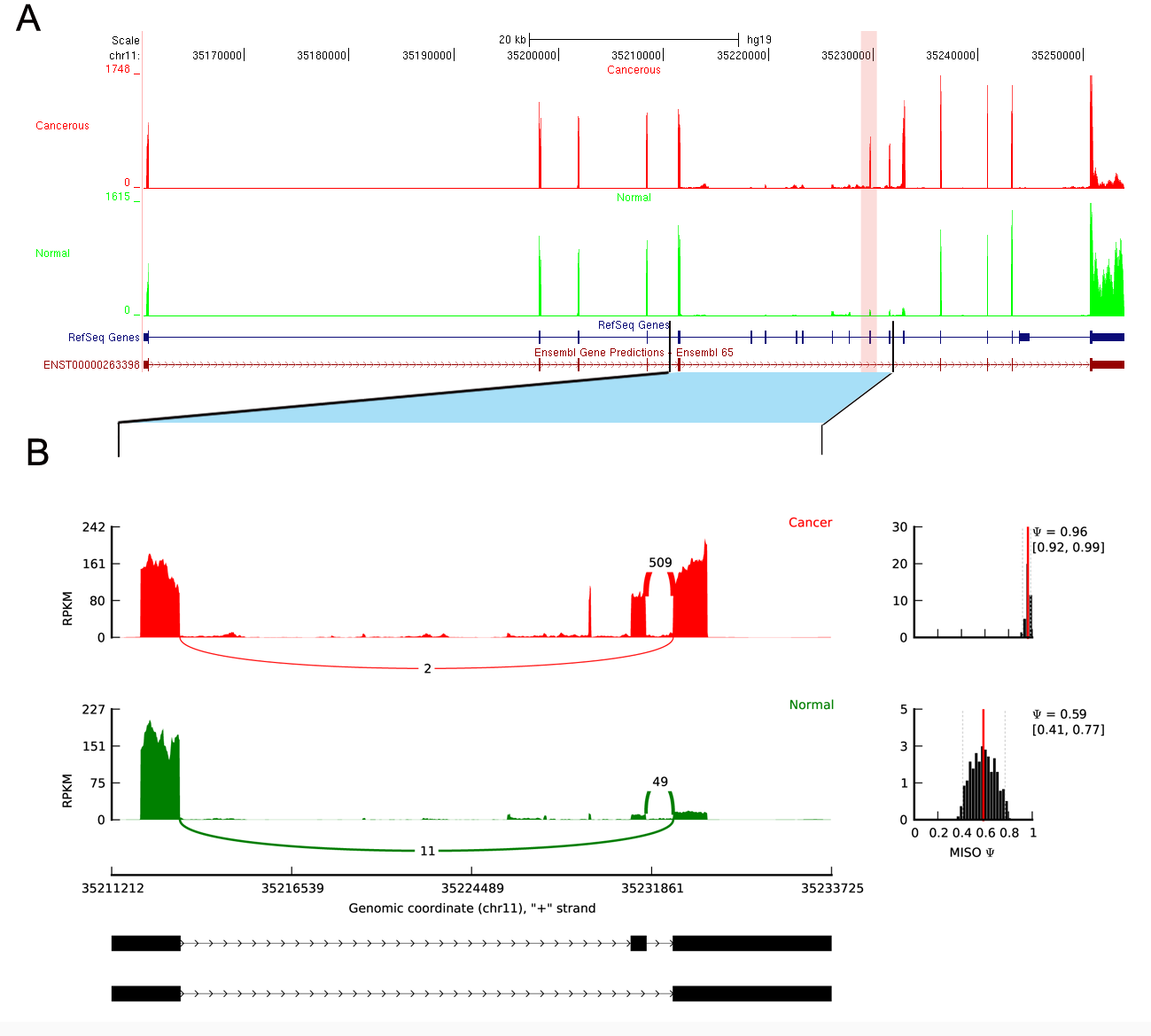

Supplement: Figure S4 — RNA-Seq read mapping to the reference gene CD44. A: RNA-Seq read mapping to the UCSC reference genome (hg19) of the gene PDGFA for UCB and normal tissues in this study. The UCB tracks are shown in red and normal tissue in green. The pink band indicated the location of skipped exon. B: The detail of junction reads mapping to the skipped exon and its neighboring exons. The Ψ (”percentage spliced in”) indicates the ratio of reads supporting inclusion exon vs. total reads supporting both inclusion and exclusion exon. The Ψ posterior distributions were shown in the right side. (TIF) [file pone.0091466.s004.tif]

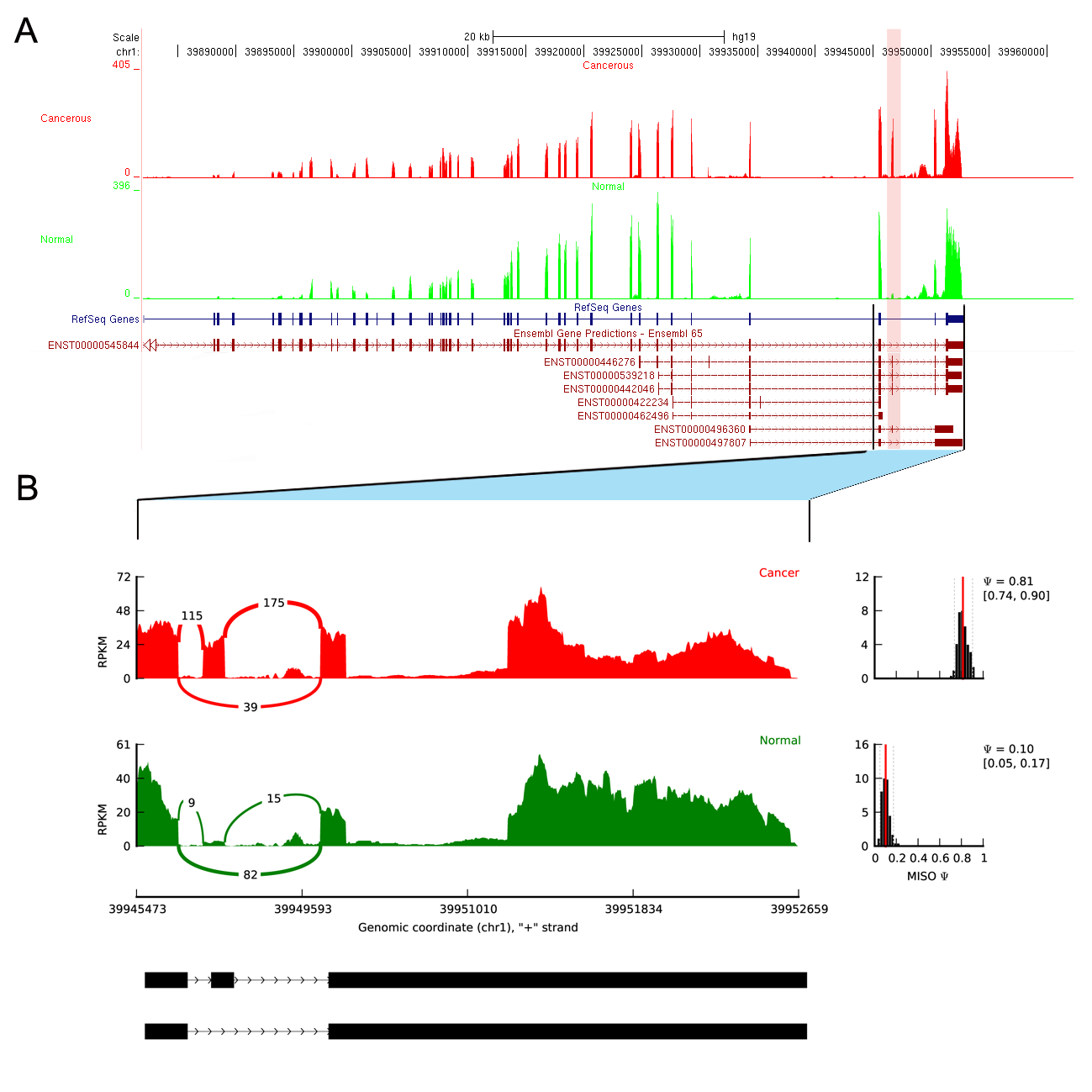

Supplement: Figure S5 — RNA-Seq read mapping to the reference gene MACF1. A: RNA-Seq read mapping to the UCSC reference genome (hg19) of the gene MACF1 for UCB and normal tissues in this study. The UCB tracks are shown in red and normal tissue in green. The pink band indicated the location of skipped exon. B: The detail of junction reads mapping to the skipped exon and its neighboring exons. (TIF) [file pone.0091466.s005.tif]

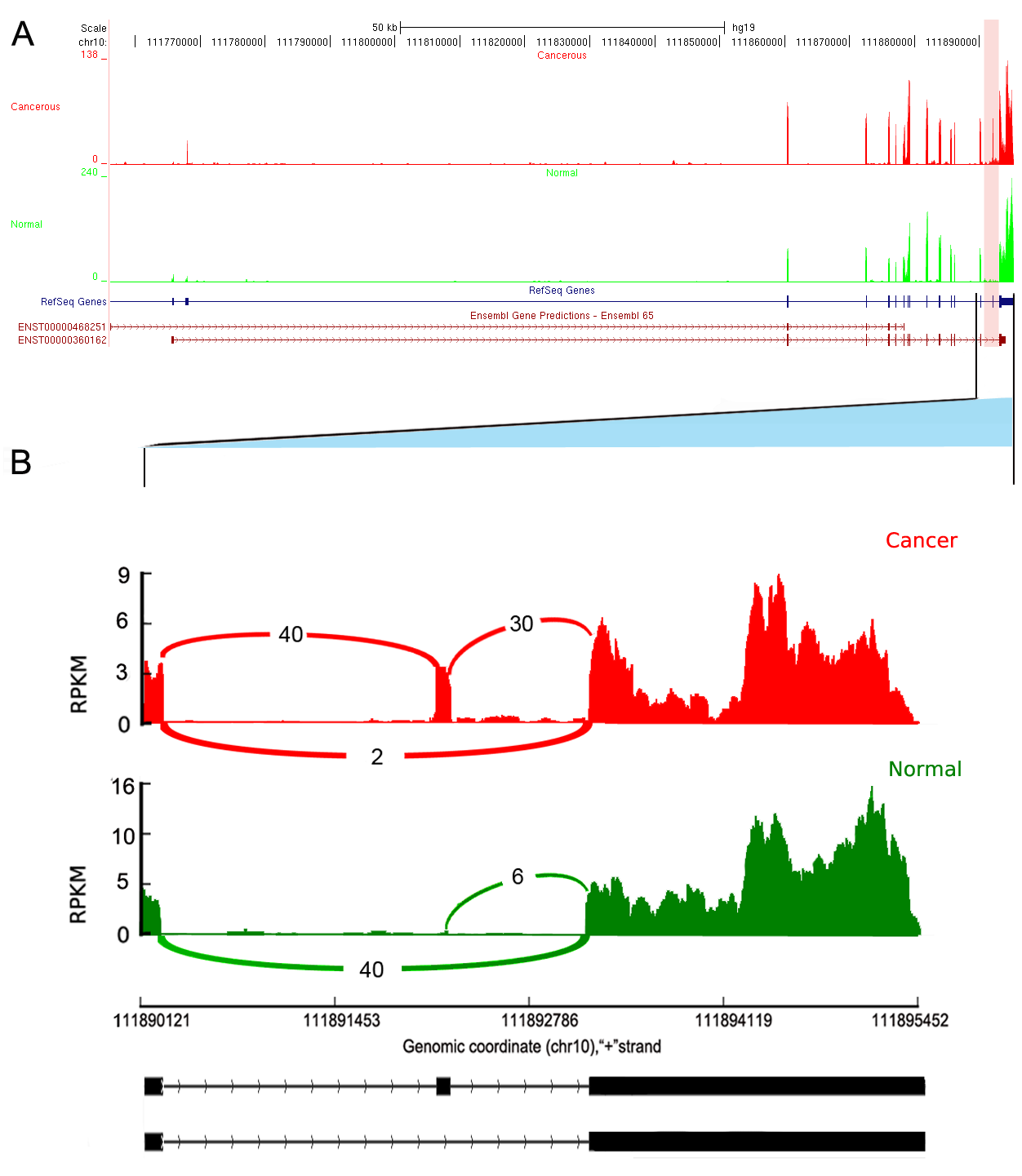

Supplement: Figure S6 — RNA-Seq read mapping to the reference gene ADD3. A: RNA-Seq read mapping to the UCSC reference genome (hg19) of the gene ADD3 for UCB and normal tissues in this study. The UCB tracks are shown in red and normal tissue in green. The pink band indicated the location of skipped exon. B: The detail of junction reads mapping to the skipped exon and its neighboring exons. (TIF) [file pone.0091466.s006.tif]

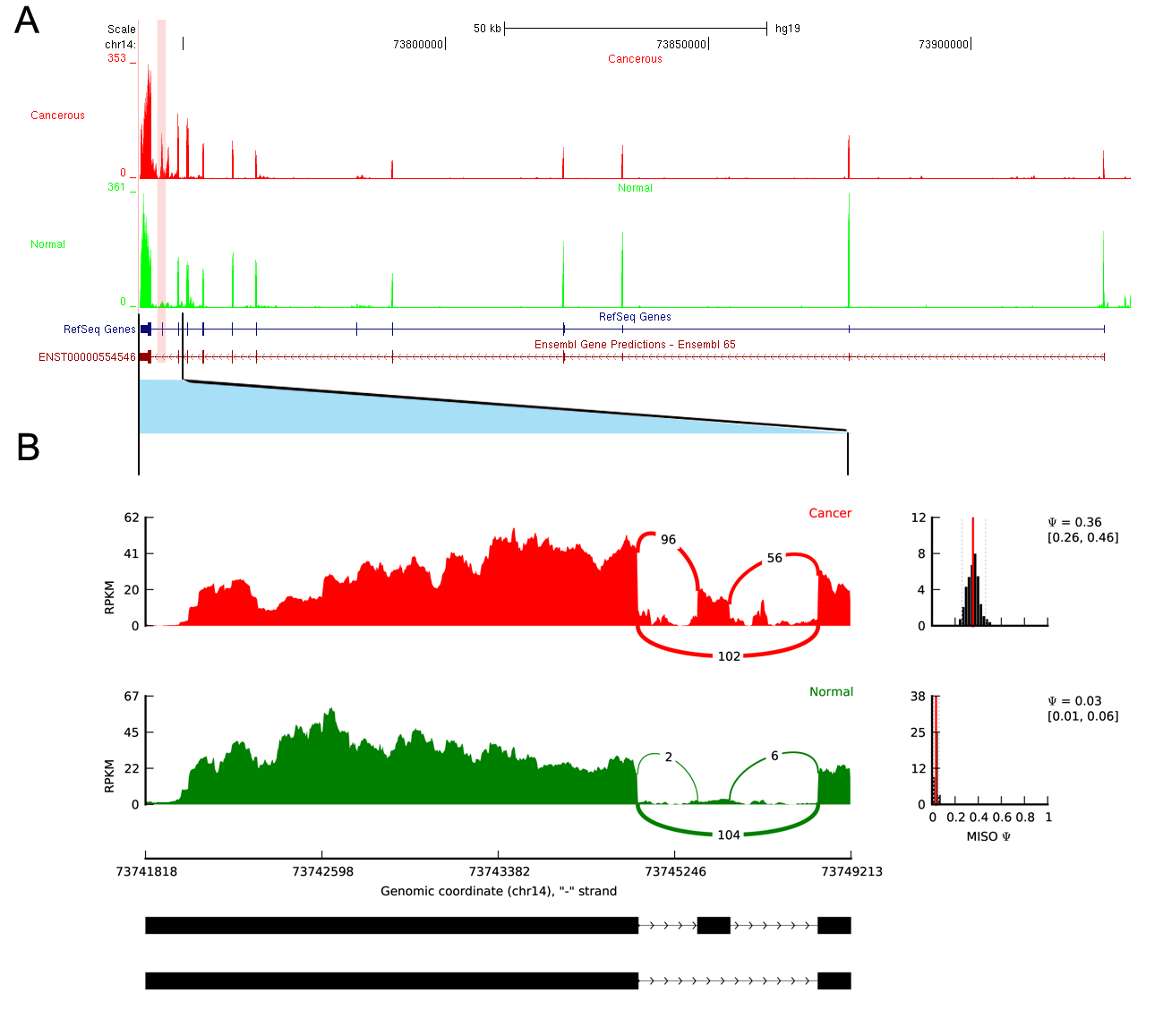

Supplement: Figure S7 — RNA-Seq read mapping to the reference gene NUMB. A: RNA-Seq read mapping to the UCSC reference genome (hg19) of the gene NUMB for UCB and normal tissues in this study. The UCB tracks are shown in red and normal tissue in green. The pink band indicated the location of skipped exon. B: The detail of junction reads mapping to the skipped exon and its neighboring exons. (TIF) [file pone.0091466.s007.tif]
